# Polyglutamine Expansion Mutation Yields a Pathological Epitope Linked to Nucleation of Protein Aggregate: Determinant of Huntington's Disease Onset

**DOI:** 10.1371/journal.pone.0000635

**Published:** 2007-07-25

**Authors:** Keizo Sugaya, Shiro Matsubara, Yasuhiro Kagamihara, Akihiro Kawata, Hideaki Hayashi

**Affiliations:** Department of Neurology, Tokyo Metropolitan Neurological Hospital, Tokyo, Japan; Laboratory of Neurogenetics, National Institutes of Health, United States of America

## Abstract

Polyglutamine (polyQ) expansion mutation causes conformational, neurodegenerative diseases, such as Alzheimer's and Parkinson's diseases. These diseases are characterized by the aggregation of misfolded proteins, such as amyloid fibrils, which are toxic to cells. Amyloid fibrils are formed by a nucleated growth polymerization reaction. Unexpectedly, the critical nucleus of polyQ aggregation was found to be a monomer, suggesting that the rate-limiting nucleation process of polyQ aggregation involves the folding of mutated protein monomers. The monoclonal antibody 1C2 selectively recognizes expanded pathogenic and aggregate-prone glutamine repeats in polyQ diseases, including Huntington's disease (HD), as well as binding to polyleucine. We have therefore assayed the *in vitro* and *in vivo* aggregation kinetics of these monomeric proteins. We found that the repeat-length-dependent differences in aggregation lag times of variable lengths of polyQ and polyleucine tracts were consistently related to the integration of the length-dependent intensity of anti-1C2 signal on soluble monomers of these proteins. Surprisingly, the correlation between the aggregation lag times of polyQ tracts and the intensity of anti-1C2 signal on soluble monomers of huntingtin precisely reflected the repeat-length dependent age-of-onset of HD patients. These data suggest that the alterations in protein surface structure due to polyQ expansion mutation in soluble monomers of the mutated proteins act as an amyloid-precursor epitope. This, in turn, leads to nucleation, a key process in protein aggregation, thereby determining HD onset. These findings provide new insight into the gain-of-function mechanisms of polyQ diseases, in which polyQ expansion leads to nucleation rather than having toxic effects on the cells.

## Introduction

To date, nine polyglutamine (polyQ) diseases have been identified: Huntington's disease (HD), spinal and bulbar muscular atrophy, spinocerebellar ataxia (types 1, 2, 3, 6, 7, and 12) and dentatorubral-pallidoluysian atrophy, each of which results from an abnormally increased number of residues in a polyQ tract of the corresponding gene product [1]. The monoclonal antibody (mAb) 1C2 has been found to selectively discriminate among critical polyQ lengths [2], [3]. Since the increased length of polyQ proteins has been associated with earlier onset and more severe manifestation of the disease state, expansion of the polyQ tract is thought to be the key causal element of the disease process [4]. PolyQ diseases have been found to belong to a wide range of neurodegenerative diseases associated with protein misfolding and aggregation, including Alzheimer's, prion and Parkinson's diseases [5], [6]. In many of these conditions, protein deposition involves the formation of amyloid fibrils, and polyQ aggregates show many of the attributes of amyloid [7], [8]. Although the role of aggregation and fibril formation in these disorders has not yet been established, protein misfolding and aggregation are thought to be the central issues for understanding the molecular mechanisms of polyQ pathogenesis [4]. Amyloid fibril growth is considered to be controlled by nucleated growth polymerization, a two-stage process consisting of the energetically unfavorable formation of a nucleus, followed by efficient elongation of the nucleus via sequential additions of monomer [9], [10].

Recent analysis of the *in vitro* aggregation kinetics of a series of polyQ peptides showed that polyQ aggregation was also due to a nucleated growth polymerization reaction [11]. Moreover, the repeat-length-dependent nucleation process of polyQ aggregation was found to reflect the length related age-of-onset of HD. The molecular bases of the relationship between repeat length and age-of-onset and between polyQ expansion and protein aggregation, however, are still unclear. Since the ability of the mAb 1C2 to detect huntingtin also depends on the length of polyQ tracts, we tested whether the nucleation process is related to the pathological epitope detected by 1C2. Based on our findings, we have hypothesized an amyloid-based polyQ pathogenic pathway that can explain most of the features characteristic of polyQ diseases. These include protein aggregate, threshold polyQ length, delayed disease-onset, repeat-length related age-of-onset and selective loss of neurons [1], [4], [12].

## Results

### Aggregation lag times and anti-1C2 signal intensity of polyQ expansions

**Figure 1:**
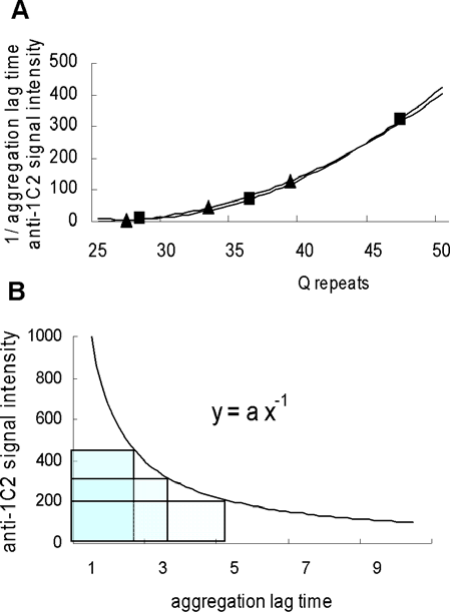
Aggregation lag times and anti-1C2 signal intensity of polyQ expansions. (A). Inverted values of the repeat-length-dependent differences in aggregation lag times of a series of polyQ peptide encoding repeats length of 28, 36 and 47 (Q28, Q36 and Q47) by Chen et al. [11] (closed rectangles) and length-dependent intensity differences of anti-1C2 signals on soluble monomers of huntingtin encoding repeats length of 27, 33 and 39 (Q27, Q33 and Q39) by Trottier et al. [2] (closed triangles). The Spearman's correlation coefficient was 1.00, with the relationship represented by the function, y = 0.72(x−25)^2^−2.75(x−25)+7.1. (B). Relationship between aggregation lag times of polyQ tracts and intensity of anti-1C2 signal on soluble monomers of huntingtin from the results of Figure 1A (y = ax^−1^, a is relative value). Each of the rectangles shows a constant area ( = a).

The relative inverted values of the aggregation lag times of polyQ peptides (Q28, Q36 and Q47) [11] were determined (Figure 1A). The intensity of the anti-1C2 signal on soluble monomers of huntingtin containing variable lengths of polyQ tracts [2] was adjusted relative to the intensity of anti-huntingtin mAb. Surprisingly, both independent measurements of the inverted values of the repeat-length-dependent differences in aggregation lag times of polyQ tracts and the length-dependent intensity differences of the anti-1C2 signal on polyQ tracts were identical (Figure 1A). Furthermore, the relationship between aggregation lag times and the intensity of the anti-1C2 signal was represented by the function, y = ax^−1^ (a is the relative value), with each of the rectangles having a constant area (Figure 1B), suggesting that the length-dependent differences in aggregation lag times of polyQ tracts are related to the integration of the length-dependent intensity of the anti-1C2 signal on soluble polyQ monomers. In agreement with this observation and in contrast to conventional models of nucleated growth polymerization, the number of monomeric units comprising the critical nucleus of polyQ aggregation was equal to 1 [11], suggesting that the rate-limiting step in the nucleation process of polyQ aggregation involves folding within the monomer.

### Aggregation lag times and anti-1C2 signal intensity of polyL expansions

**Figure 2:**
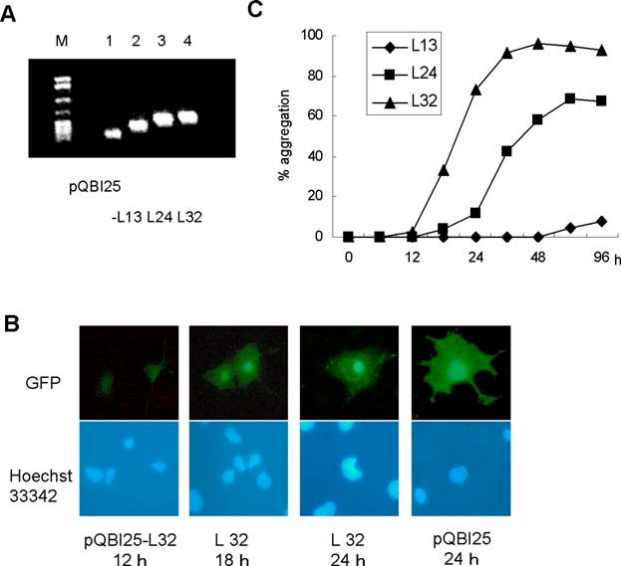
Time course of aggregate formation by cells transfected with constructs expressing variable lengths of polyL-GFP fusion proteins. (A). Agarose gel electrophoresis of PCR products amplified by a set of primers covering the inserted fragments of the constructs (Lane M, pBR322 digested with MspI as size marker; lane 1, pQBI25; lane 2, pQBI25-L13; lane 3, pQBI25-L24; lane 4, pQBI25-L32), and yielding PCR of sizes 153 bp, 228 bp, 261 bp, and 285 bp, respectively. Migration of the PCR products was slightly different in proportion to the number of CTG repeats. (B). Fluorescence micrograph of the expressed GFP or polyL-GFP fusion protein. Nuclei were stained with Hoechst 33342. (C). Summary of the time course of aggregation of cells transfected with pQBI25-L13, L-24 and -L32. Percent aggregation represents the number of cells with aggregates relative to the number of GFP-positive cells at each time point. Values represent the mean of duplicate experiments.

**Figure 3:**
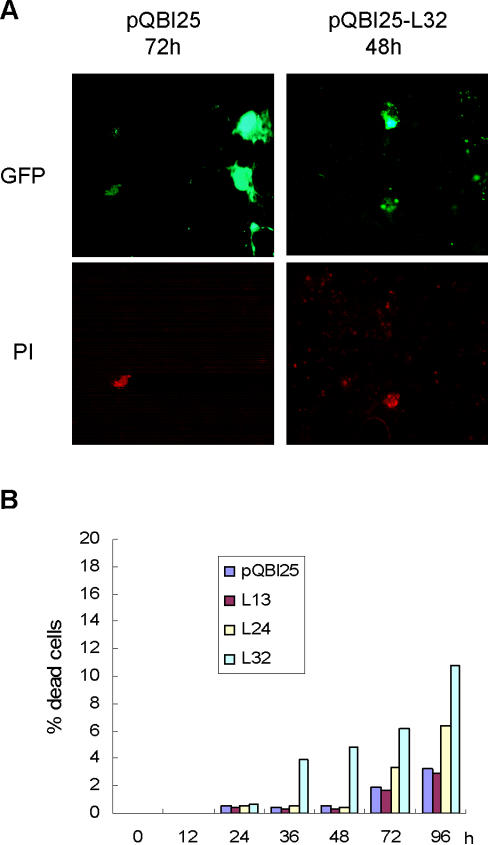
Detection of dead cells induced by polyL-GFP fusion proteins. (A). Fluorescence micrograph of propidium iodide (PI) stained dead cells expressing GFP or polyL32-GFP fusion protein. (B). Summary of the time course of cell death following transfection with pQBI25, pQBI25-L13, -L24 and -L32. The percentage of dead cells is reported relative to the number of GFP-positive cells at each time point transfection. Values represent the mean of duplicate experiments.

Polyleucine (polyL) tracts can be detected by 1C2, as well as displaying a higher propensity for aggregation and toxicity in cells compared to polyQ tracts [13]. To confirm the relationship between the nucleation process of protein aggregation and the pathological epitope detected by 1C2, we made constructs in which variable lengths of polyL were fused to the N terminus of green fluorescent protein (GFP) (Figure 2A). COS7 cells expressing a series of polyL-GFP fusion proteins (L13, L24 and L32) and GFP alone were serially observed for 120 h, and the numbers of transfected cells with and without visible aggregate formation were counted. When we assessed the time course of aggregate formation in COS7 cells transfected with pQBI25-L32 or vector alone, we found that, in contrast to cells transfected with vector alone (pQBI25, 24 h), cells transfected with pQBI25-L32 show aggregates of fusion protein in their nuclei (L32, 24 h) (Figure 2B). The fraction of cells containing aggregates decreased 48 h after transfection with pQBI25-L32 and 72 h after transfection with pQBI25-L24 (Figure 2C), probably due to cell death caused by aggregate formation. To clarify the effect of cell death due to polyL expansion on aggregate formation, we serially monitored dead cells by propidium iodide (PI) staining for 96 h after transfection with pQBI25, pQBI25-L13, -L24 or -L32 (Figure 3A). In contrast with cells transfected with pQBI25, a small percent of GFP-positive cells died 36 h after transfection with pQBI25-L32 and 72 h after transfection with pQBI25-L24 (Figure 3B). The percentage of dead cells in cultures transfected with pQBI25 or pQBI25-L13 remained constant over 96 h after transfection.

**Figure 4:**
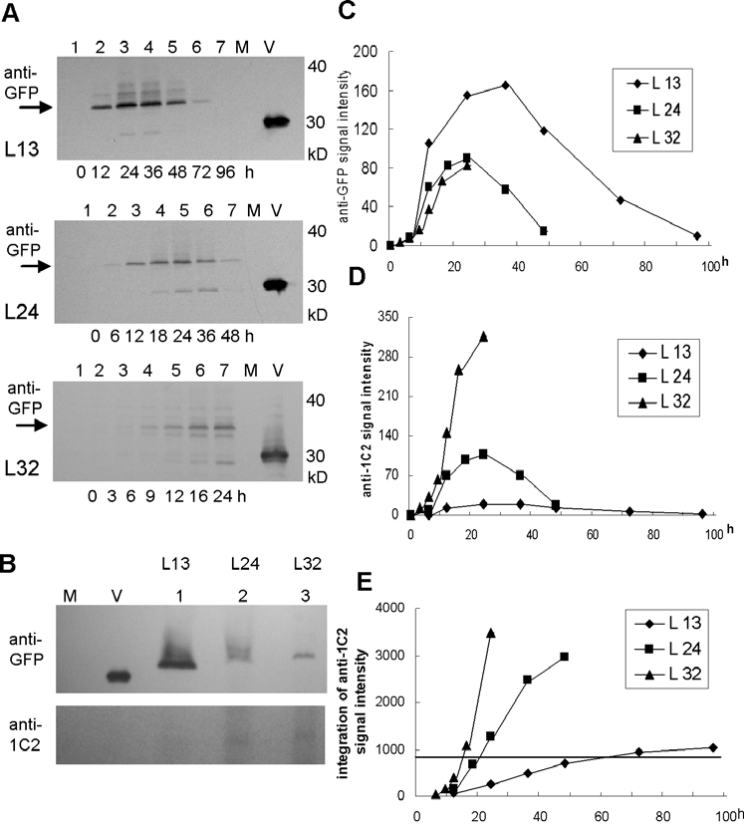
Expression of epitopes binding anti-GFP and anti-1C2 on soluble monomers of polyL-GFP fusion proteins. (A). Time course of anti-GFP Ab binding of cells expressing polyL-GFP fusion proteins (M, lysate from mock transfected cells; V, lysate from cells transfected with the pQBI25 vector). (B). Western blot analysis of GFP and polyL-GFP fusion proteins successively immunoprobed with 1C2 and anti-GFP Ab. (C). Expression of anti-GFP signal on polyL-GFP fusion proteins. The signal intensity of each band on Western blots (Figure 4A) was adjusted to be equal to that shown in Figure 2C. Values represent mean of duplicate experiments. (D). Expression of anti-1C2 signal on the polyL-GFP fusion proteins L13, L24 and L32. The relative signal intensities of each was determined by the results of Figure 4B, and the time course of anti-1C2 signal intensity was calculated from the data in Figure 4C, since anti-1C2 signal intensity of 13 leucine fused to GFP was weak. (E). Integration of anti-1C2 signal intensity on polyL-GFP fusion proteins, calculated from the results of Figure 4D using Simpson's formula. Spearman's correlation coefficients calculated from the data in Figure 2C and Figure 4E were, for L13, R = 0.821 (p = 0.025); for L24, R = 0.973 (p = 0.009); and for L32, R = 0.986 (p = 0.014).

Cells transfected with each construct, as well as mock transfected cells, were serially collected after transfection and analyzed by Western blotting using anti-GFP polyclonal antibody (Figure 4A). To exclude the effect of dead cells due to polyL expansion, cells transfected with pQBI25-L32 and pQBI25-L24 were monitored for only 24 and 48 h, respectively (Figure 3). Equal amounts of protein lysates obtained from cells 24 h after transfection were analyzed using 1C2 and anti-GFP antibody (Figure 4B), with the intensity of each signal adjusted to that of the sum of the constant squares as in Figure 2C. The time courses of the intensity of the anti-GFP signal (Figure 4C) and anti-1C2 signal (Figure 4D) were then plotted, with the integration of intensity of the anti-1C2 signal at each time point calculated by Simpson's formula. Remarkably, the integration of the repeat-length-dependent intensity of the anti-1C2 signal (Figure 4E) was consistent with the time course of formation of the length-dependent aggregates (Figure 2C, p<0.05). These results suggest that the repeat-length-dependent differences in aggregation lag times of polyL-GFP fusion proteins are related to the integration of the length-dependent intensity of the anti-1C2 signal on soluble monomers of polyL-GFP fusion proteins.

### Critical determinant of the repeat-length dependent age-of-onset of HD

**Figure 5:**
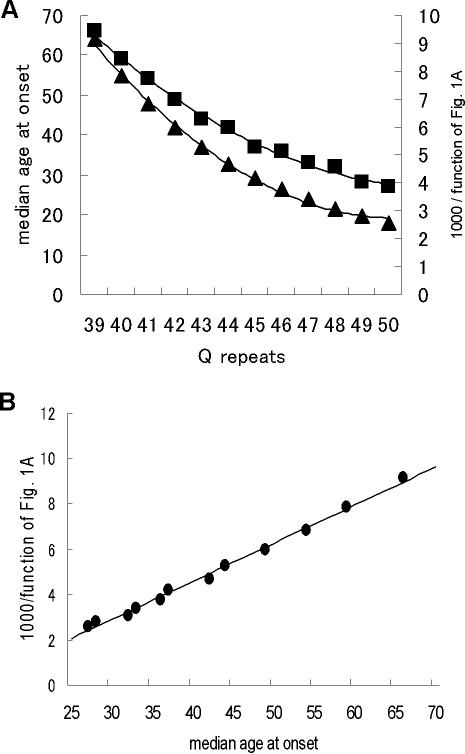
Determinant of HD age-of-onset. (A). Median age at onset of HD relative to particular repeat size. Data were obtained from a large cohort study consisting of 661 affected and 205 asymptomatic individuals at risk [14] (rectangles). The median age at onset is the age at which 50% of individuals will be affected. Inverted values of the quadratic function, y = 0.72(x−25)^2^−2.75(x−25)+7.1, were obtained from the results in Figure 1A (triangles). Spearman's correlation coefficient was 1.00, p = 0.00046. (B). Correlation between the quadratic function and the median age at onset of HD relative to repeat size, from the results of Figure 5A (circles).

While the ability of 1C2 to detect the pathological epitope is associated with the nucleation process of protein aggregates, it is unclear whether this association reflects the process of polyQ pathogenesis. We therefore assessed the relationship between the function obtained from the results of Figure 1A and the repeat-length dependent age-of-onset of HD patients. Results from a large cohort, consisting of 661 affected and 205 asymptomatic at-risk persons with polyQ expansion, showed that median age of HD onset was related to CAG (glutamine) size [14] (Figure 5A). Surprisingly, the inverted values of the quadratic function from the correlation between the aggregation lag times of polyQ tracts and the intensity of anti-1C2 signal on soluble monomers of huntingtin perfectly reflected the repeat-length dependent age-of-onset of HD (Figure 5A, Spearman's correlation coefficient = 1.00, p<0.001). Despite the complexity of the cellular environment, including degradation and transport processes capable of partitioning proteins into different molecular forms and compartments, and the presence of chaperones that modulate polyQ aggregation and cellular toxicity [15], [16], our results strongly suggest that the pathological epitope detected by 1C2 and its link to nucleation are critical in determining HD onset. These findings also demonstrated that an additional factor, dependent on the repeat-length of polyQ expansions or associated with aging brain, participates in determining HD onset (Figure 5B). It is of interest that the expression of huntingtin associated protein-1, a strong candidate for involvement in HD pathology [17], [18], decreases in the aging brain at the main pathological sites of HD, including the caudate putamen, globus pallidus and neocortex [19].

## Discussion

Since the discovery of the androgen receptor gene mutation in the polyQ diseases spinal and bulbar muscular atrophy [20], the increased knowledge of various polyQ diseases has shown that the unifying pathogenic mechanism of these diseases and of their characteristic features arises from the expansion of polyQ itself [1], [4], [12], [21]. To date, however, there has been no hypothetical mechanism that can explain all of the features of these diseases. In particular, an initial process directly linked to the pathogenic mechanisms has not yet been discovered. The results presented here show that the repeat-length-dependent differences in aggregation lag times of variable lengths of polyQ and polyL tracts are strictly linked to the length-dependent epitope detected by the mAb 1C2, which selectively discriminates among the pathological lengths of glutamine repeats in polyQ diseases [2], [3]. Strikingly, the correlation between the inverted values of aggregation lag times of polyQ tracts and the intensity of anti-1C2 signals on soluble monomers of huntingtin precisely reflects the repeat-length dependent age-of-onset of HD patients. Consistent with our findings, the rate-limiting nucleation process of polyQ aggregation is thought to involve folding within mutant monomers [11]. These data suggest that the protein surface structure detected by 1C2 in soluble mutant monomers acts as an amyloid-precursor epitope, leading to nucleation, a key process of protein aggregation, and thereby determining HD onset. Moreover, 1C2 has been shown to inhibit the *in vitro* aggregation of the protein implicated in HD [22].

**Figure 6:**
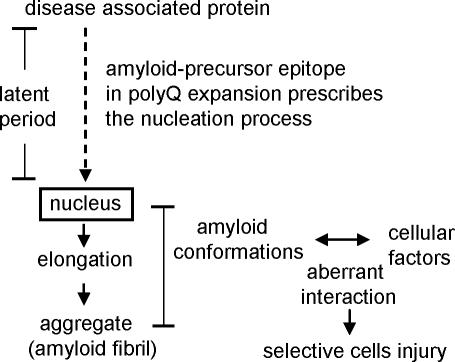
Proposed pathway of amyloid-based polyQ pathogenesis. The two step process of the gain-of-function mechanisms of polyQ pathogenesis are shown. The polyQ expansion mutation results in a gain of the amyloid-precursor epitope, leading to the nucleation process of polyQ aggregation. During the nucleated growth polymerization reaction, the diversity of amyloid conformations results from the protein's primary structure containing the polyQ segment. Aberrant interactions between amyloid conformations and cellular factors determine the toxic effects on the cells, resulting in selective injury.

Our results indicate that the gain-of-function of polyQ pathogenesis involves two steps. A gain-of-toxic-function mechanism for polyQ expansion mutation has been suggested by results from cell transfection and transgenic and knock-out animal experiments [12], [23]. Our findings suggest, however, that the genetic gain-of-function conferred by polyQ expansion is a gain of amyloid-precursor structure rather than a toxic effect on the cells. This scenario may explain the puzzling relationship between protein aggregation and cell toxicity, in that aggregate formation does not necessarily result in cell death. For example, aggregates have been detected in the dentate nucleus of the HD cerebellum, a brain region unaffected in this disease, and a cellular model has shown a discrepancy between aggregate formation and cell death [3], [24]. In contrast, in both polyQ transgenic mice and Drosophila, interference with aggregate formation has been shown to prolong survival and to ameliorate neuropathology [25] [Bibr pone.0000635-Sanchez1]–[27]. These results suggest that the process of aggregate formation is necessary for, but does not necessarily result in, cell toxicity. Correspondingly, the genetic gain-of-function of polyQ expansion leads to the nucleation process of aggregate formation, but does not have a direct toxic effect on the cells. Our results, together with recent findings in conformational diseases, have led us to propose an amyloid-based polyQ pathogenic pathway (Figure 6). Remarkably, this basic pathway can explain most of the characteristic features of polyQ diseases that are due to polyQ expansion.

Although a lower range of polyQ expansion is a normal polymorphism, at a higher range, or threshold polyQ length, partial conversion of a structure linked to nucleation of protein aggregates can trigger an amyloid-precursor state, leading to the pathogenic pathway of polyQ diseases. The neurological symptoms of polyQ diseases typically appear in midlife. The structural conversion from the precursor protein to the nucleus is an energetically unfavorable reaction, requiring a very long time under physiological conditions [11]. This delayed disease-onset can be considered a latent period. Compared with other conformational diseases, the most characteristic feature of polyQ diseases is the relationship of age-of-onset to repeat length. The present study demonstrated that a unique structural feature of the dependency of amyloid-precursor epitope on length of polyQ expansions and its link to nucleation are the critical determinants of the repeat-length related age-of-onset of HD.

In polyQ diseases, there is a selective loss of neurons, with different cells showing different levels of vulnerability. A common feature of amyloid-forming proteins is that a single protein can adopt multiple distinct, self-propagating amyloid conformations, with the spectrum of misfolded forms being determined by the protein's primary structure [28]–[30]. Amyloid fibril formation of yeast prion protein Sup35 also occurs by nucleated growth polymerization [31]. This pathway yields conformational variants of Sup 35, resulting in strain differences in yeast prion phenotype [31], [32]. Although this conformational variation is still undetermined in polyQ diseases, specific amyloid conformations adopted by each protein containing polyQ segments can affect different cellular factors including proteins, thereby modulating the toxic effects. Conformational diversity and selective interaction of amyloid conformations with cellular factors may determine the toxic effects on cells, resulting in selective loss of neurons and phenotypic variations.

## Materials and Methods

### Plasmid construction

Genomic DNA was extracted from whole blood by standard procedures after informed consent. Truncated exon 1 of the human HD gene, including polymorphic CAG repeats, was amplified by PCR using the primers 5′-atgaaggccttcaagtccctcaag-3′ and 5′-ggcggtggcggctgttgctgctgc-3′. The purified PCR fragment was inserted into the Nhe1 site of the GFP expression vector, pQBI25 (Takara, Japan), in both directions and used to transform competent *E*. *coli* according to the manufacturer's protocol. Positive clones were selected by the PCR method, and all constructs were validated by DNA sequencing (Takara customer service). This yielded a series of fusion proteins of polyQ-GFP and polyL-GFP encoding repeat lengths of 13, 24 and 32 (L13, L24 and L32). All constructs were transfected into COS7 cells and observed for 120 h. We confirmed that such a lower expansion of polyQ tracts never formed visible aggregates (data not shown).

### Cell culture and transfection

COS7 cells were obtained from the Riken Cell Bank (Japan) and maintained in Dulbecco's modified Eagle's medium (Nissui Pharmaceutical, Japan) supplemented with 10% fetal bovine serum. For transfection of plasmid DNA, cells were seeded at 1.5×10^5^ cells/35 mm plate, overlain with a sterile coverslip, and grown to 70–80% confluence. The cells were washed twice with Opti-MEM (Invitrogen) and transfected with 1.5 µg plasmid DNA in Lipofectamine reagent (Invitrogen) for 6 h according to the manufacturer's instructions.

### Analysis of aggregation *in vivo*

COS7 cells transfected with pQBI25-L13, -L24 or -L32 or vector alone, pQBI25, were washed with phosphate-buffered saline without Ca^2+^ and Mg^2+^ (PBS(-)) and fixed with 4% paraformaldehyde for 30 min at room temperature. The cells were washed with PBS(-), stained with 10 uM Hoechst 33342 for 15 min at room temperature, washed again with PBS(-) and mounted with Slowfade Gold antifade reagent (Invitrogen). Cells were serially examined by fluorescence microscopy until 120 h after transfection. The number of transfected cells with visible aggregates and the number of transfected cells without aggregates were counted independently in randomly chosen microscopic fields, with approximately 300–500 transfected cells analyzed per experiment.

### Analysis of dead cells

Dead cells were detected using Live-Dead Cell Staining Kit (Biovision), which stains dead cells with PI, a cell non-permeable red fluorescent dye. The cells were incubated with Staining Buffer for 15 min at 37°C, mounted with Slowfade Gold antifade reagent, and examined by fluorescence microscopy until 96 h after transfection. The number of transfected cells with or without PI signal was counted in randomly chosen microscopic fields, with approximately 300–400 transfected cells analyzed per experiment.

### Western blotting analysis

In each experiment, 9.6 µg plasmid DNA were transfected into COS7 cells on 100 mm plates; this represents an equal ratio of plasmid DNA per square in fluorescence microscopy. Cells were serially collected after transfection and lysed by homogenization in 50 mM Tris-HCl pH 8.0, 10% (v/v) glycerol, 5 mM EDTA, 150 mM KCl, 1 mM PMSF. Insoluble materials were removed by centrifugation at 10,000×g at 4°C for 10 min, and the protein concentration of each supernatant was determined using the Bradford procedure. To determine the time course of expression of each construct, the amount of applied protein was adjusted in proportion to the total amount of cellular protein in each sample, starting with 25 µg of cellular protein at 0 h post-transfection. Samples were electrophoresed on 7.5% SDS-polyacrylamide gels and transblotted to nitrocellulose membranes, which were blocked with 5% non-fat dry milk. Each membrane was incubated with horseradish peroxidase-conjugated anti-GFP polyclonal Ab (Rockland) diluted 1∶50,000. Equal amounts of protein lysates (50 µg) from mock transfected cells and from cells transfected with pQBI25-L13, -L24 or -L32, and 15 µg of protein lysate obtained from cells 24 h after transfection with pQBI25 were analyzed on the same blot using 1C2 (Chemicon) diluted 1∶2,000. Each membrane was incubated with horseradish peroxidase-conjugated anti-mouse IgG (Santa Cruz) diluted 1∶1000 dilution, stripped and reprobed with anti-GFP Ab. Proteins were detected using the enhanced chemiluminescence (ECL) detection system (Amersham Biosciences), and signal intensity was measured by NIH Image software.
